# Technical Advancements and Applications in Predictive Modeling of Polyurethane Foaming Height

**DOI:** 10.3390/polym17040452

**Published:** 2025-02-08

**Authors:** Chil-Chyuan Kuo, Yi-Qing Lu, Armaan Farooqui, Song-Hua Huang

**Affiliations:** 1 Department of Mechanical Engineering, Ming Chi University of Technology, No. 84 Gungjuan Road, New Taipei City 24301, Taiwan; 2 Research Center for Intelligent Medical Devices, Ming Chi University of Technology, No. 84 Gungjuan Road, New Taipei City 24301, Taiwan; 3 Department of Mechanical Engineering, Chang Gung University, No. 259 Wenhua 1st Rd., Guishan Dist., Taoyuan City 33302, Taiwan; 4 Center for Reliability Engineering, Ming Chi University of Technology, No. 84 Gungjuan Road, Taishan District, New Taipei City 24301, Taiwan; 5 Department of Mechanical Engineering, Chhattisgarh Swami Vivekanand Technical University, Bhilai 491107, Chhattisgarh, India; 6 Li-Yin Technology Co., Ltd., No. 37, Lane 151, Section 1, Zhongxing Road, Wugu District, New Taipei City 24301, Taiwan

**Keywords:** polyurethane foam, foaming rate, foaming height, foaming time, simulation

## Abstract

Various polyurethane foams (i.e., rigid, flexible, and spray polyurethane foams) offer diverse applications due to their unique properties, including thermal insulation, cushioning, and seamless gap filling. These foams provide solutions across industries such as construction, automotive, and refrigeration. However, the foaming process presents several challenges that may result in various defects in the final products. This work provides innovative predictive techniques for polyurethane foam expansion and applications in advanced manufacturing processes. The foaming height of the third polyurethane foaming agent (PU-3) closely aligned with the experimentally measured values. The relationship between foaming height and time is influenced by the type and concentration of catalysts, as well as the blowing agents used. However, simulations using Moldex 3D Version 2024 revealed a nonlinear relationship between foaming height and time, characterized by three distinct foaming rates. Zone B demonstrated the highest foaming rate, followed by Zone C, while Zone A showed the lowest rate. The foaming height and rate were significantly influenced by the foaming angle, with smaller angles enhancing both parameters. At a mold temperature of 30 °C and with an expansion coefficient of 35, the predicted foaming height of the polyurethane agent achieved an average accuracy of approximately 96% across four foaming angles. Based on these experimental findings, this study introduces three mechanisms involved in the foaming process of polyurethane foam components.

## 1. Introduction

Polyurethane foaming [1] transforms polyurethane into a lightweight, porous material by incorporating gas bubbles into the polymer matrix. This process begins by mixing polyol and isocyanate with catalysts, blowing agents, and surfactants to manage the reaction and create a foam structure. The chemical reaction produces gas, often carbon dioxide or water vapor, which forms bubbles that cause the mixture to expand into foam. As the foam cures, it solidifies and can be shaped using molds or applied directly to surfaces. Polyurethane foam has three main forms: flexible, rigid, and integral skin. Flexible foam is ideal for cushioning furniture, mattresses, and automotive seats due to its softness and compressibility. Rigid foam, prized for its thermal insulation and structural integrity, is used in construction panels, refrigerator insulation, and packaging. Integral skin foam, featuring a dense outer layer and softer core, suits applications such as steering wheels and armrests. Polyurethane foam finds applications in various fields including electronics, construction, automotive, furniture, and packaging. Its advantages include being lightweight, having excellent thermal insulation and impact resistance, and having the ability to be molded into diverse shapes and sizes.

Rigid polyurethane foam components have diverse applications, including structural foam, thermal insulation, decorative panels, packaging, imitation wood, floral foam, models, and prototypes [2,3]. Sture et al. [4] created rigid PU foam parts using a fourth-generation blowing agent and reactive catalysts and showed that exposure to UV light for approximately 1000 h did not significantly damage the material’s internal cellular structure. Li et al. [5] utilized a custom-built gas explosion testing platform to investigate the flame-retardant properties of PU foam, demonstrating its excellent flame-quenching capabilities. Optimal suppression was achieved with a 20 cm long polyurethane foam filling a 1.8 cm space. Zemła et al. [6] found that incorporating 1.0 wt.% phosphorus into rigid PU foam renders it self-extinguishing. Ran et al. [7] examined the grouting mechanisms of polyurethane composite materials and discovered that geopolymer filled with PU foam pores forms a stable, consolidated body capable of withstanding loading, cutting, and machining [8,9,10,11,12]. This makes rigid PU foam suitable as a backing material for large, rapid tooling [13].

Polyurethane foam materials possess numerous advantages. However, challenges in the foaming process can lead to defects in the final products. Issues such as inconsistencies in height, density, pore number, foaming uniformity, warpage, and deformation are difficult to control during the foaming of polyurethane products [14,15,16]. To better understand the dynamic behavior of polyurethane foam materials during foaming, this study employs Moldex 3D [17,18] to simulate the foaming process and dynamics. Additionally, the mechanical properties of polyurethane foam parts are closely linked to bubble size and density [19,20,21,22]. This research uses Moldex 3D to analyze polyurethane foam parts and conducts experiments to validate the simulation results. The study also proposes key technologies for accurately predicting the height of polyurethane foam molding. The research topics include the foaming height, density, number of pores and foaming uniformity of polyurethane under different temperature environments.

## 2. Experimental Details

**Figure 1:**
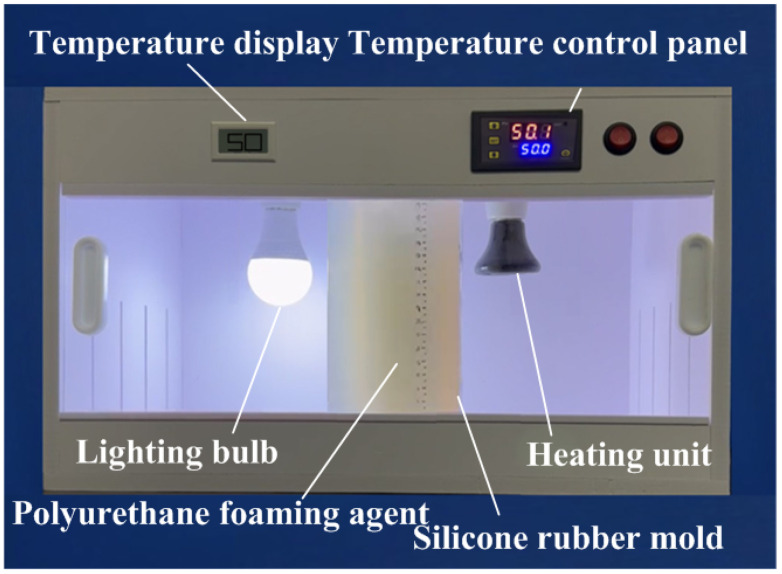
Polyurethane foam formation under temperature control within the thermostatic chamber.

**Figure 2:**
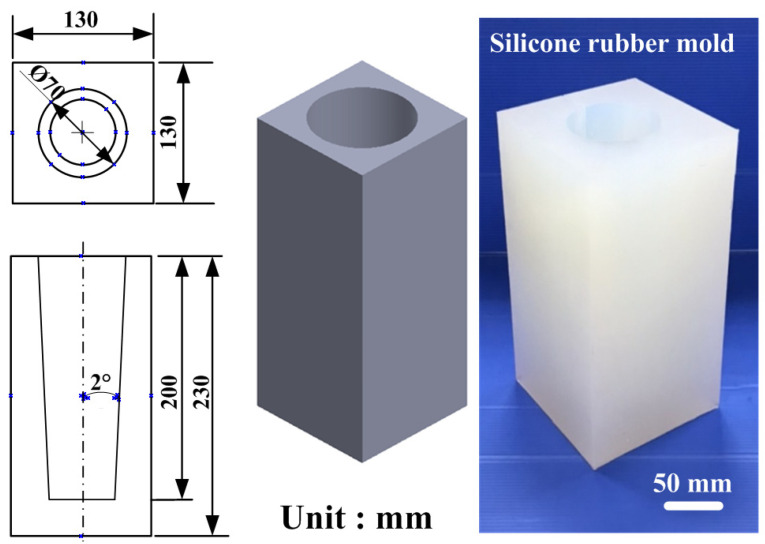
Silicone rubber mold used for polyurethane foaming.

**Figure 3:**
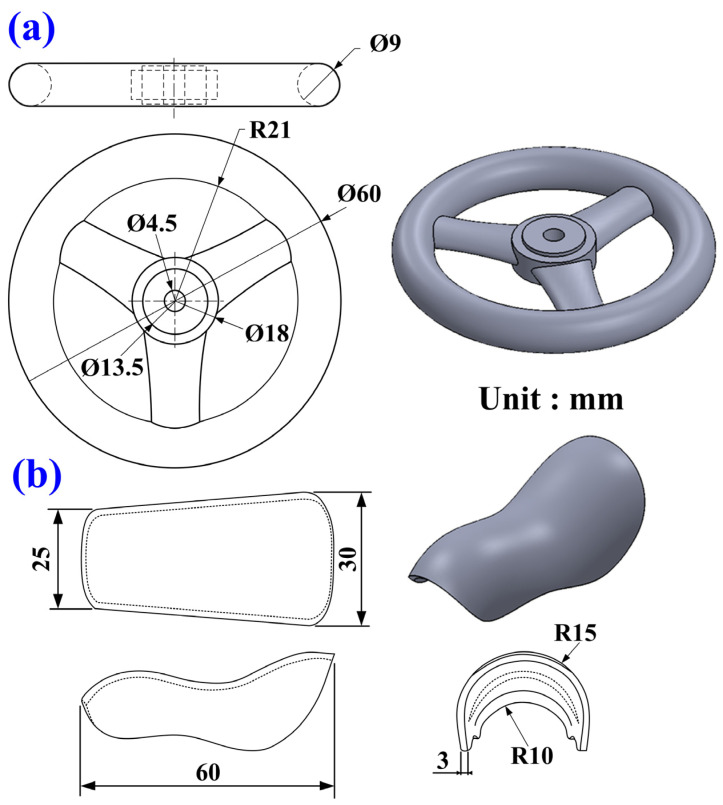
Design schematics for validation cases (**a**) one and (**b**) two of the polyurethane foaming agent.

Polyurethane foam molding is a widely employed manufacturing technique for producing lightweight, high-strength, and thermally insulating products. To explore the foaming behavior of polyurethane blowing agents at varying silicone rubber mold temperatures, silicone molds were placed inside a thermostatically controlled chamber. The internal temperature of the chamber was precisely regulated using a control panel, ensuring the molds were preheated to the target experimental temperature. Once the polyurethane blowing agent was prepared, it was poured into the preheated silicone molds to initiate the foaming process, with the foam height monitored throughout the procedure within the controlled environment. Figure 1 shows the polyurethane foam formation under temperature control within the thermostatic chamber. This research explores several key aspects: (a) analysis of polyurethane’s foaming height across five distinct mold temperatures, i.e., 20, 30, 40, 50, and 60 °C; (b) determination of the foam density under these same temperature conditions; (c) examination of the pore distribution in polyurethane foam at each temperature setting; and (d) assessment of the uniformity of the foaming process at different mold temperatures. Figure 2 depicts the silicone rubber mold used in the experiments, including its physical characteristics. The mold measures approximately 130 mm in length, 130 mm in width, and 230 mm in height. This study utilized a fused deposition modeling machine (Mark Two, Markforged Inc., Waltham, MA, USA) to print the master model using carbon fiber composite filament stocks (Onyx, Markforged Inc., Waltham, MA, USA). The primary reason for this choice is the superior surface quality and mechanical properties of the produced master models. This study utilized PU 51 foam (Puff 101 Dino Inc., New Taipei City, Taiwan) to cast parts in the silicone rubber mold, with a density of 0.64 g/cm³, a Shore hardness of 55 D, a tensile strength of 44 MPa, an elongation at break of 18%, a flexural strength of 75 MPa, and a compression strength of 84 MPa. To create the SRM, silicone rubber (KE-1310ST, Shin Etsu Inc., New Taipei City, Taiwan) and a curing agent (CAT-1310S, Shin Etsu Inc., New Taipei City, Taiwan) were mixed in a 10:1 weight ratio. The mixing process involved a vacuum machine (F-600, Feiling Inc., New Taipei City, Taiwan) to eliminate air bubbles. The vacuum process involves setting the vacuum pressure to −80 kPa and applying it for 5–10 min immediately after mixing to capture air bubbles introduced during the process. Preliminary experiments optimize these parameters to ensure effective bubble removal. The base compound (XK-019402N POLYOL, Axson Technologies Inc., New Taipei City, Taiwan) and the hardener (XK-019402 ISOCYANATE, Axson Technologies Inc., New Taipei City, Taiwan) were mixed in a weight ratio of 1:2. This study utilized a digital height gauge (Mitutoyo Inc., Shimogurimachi, Japan) to measure the height of the polyurethane foam parts. The hardness of the polyurethane foam parts was evaluated based on the ASTM D2240 standard using the Shore O surface hardness test (MET-HG-A, SEAT Inc., New Taipei City, Taiwan). This study designed two additional polyurethane foam components for testing to further validate the simulation results of the polyurethane foaming process using Moldex3D. Figure 3 shows the design schematics for validation cases 1 and 2 of the polyurethane foaming agent. The first verification specimen is a steering wheel with an outer diameter of 60 mm, an inner diameter of 42 mm, and a thickness of 9 mm. The second verification specimen is a bicycle seat with dimensions of 60 mm in length, 30 mm in width, and 30 mm in height.

## 3. Results and Discussion

To analyze the foaming behavior of the polyurethane foaming agent within the mold, this study employed Moldex3D mold flow simulation software to investigate three different polyurethane foaming agents. Table 1 outlines the parameters employed in simulating the polyurethane foaming process. The mass of the mixture injected into the silicone mold was maintained at 18 g, with a polyurethane foaming agent filling time of 4.8 s. The mass flow rate was determined to be 3.75 g/s, while the density of the resultant polyurethane foam was measured at 1.087 kg/m³. The volume flow rate reached 3.45 m³/s, and the silicone mold’s volume was specified as 626.32 cm³. The foaming process was allowed to proceed for 180 s. Mold temperatures were varied across five distinct settings: 20 °C, 30 °C, 40 °C, 50 °C, and 60 °C, while the ambient temperature was consistently maintained at 25 °C. The equations used to calculate mass flow rate, filling volume flow rate, and foaming time are shown in Equations (1), (2), and (3), respectively. In these equations, W, t_1_, Q_w_, ρ, Q_v_, V, and t_2_ represent the total weight of the polyurethane foaming agent, the filling time, mass flow rate, material density, volume flow rate, mold cavity volume, and foaming time, respectively.

Q_w_ = W/t_1_ (1)

Q_v_ = Q_w_/ρ (2)

t_2_ = V/Q_v_ (3)

**Figure 4:**
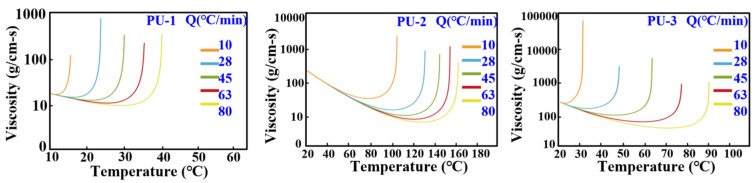
Properties of the three polyurethane foaming agents used in this study.

To ensure that the simulation results align closely with experimental outcomes, material selection is a critical factor influencing the accuracy of simulations. This study utilized three different types of polyurethane foaming agents in the Moldex3D mold flow analysis software to investigate their foaming characteristics. Figure 4 illustrates the properties of the three polyurethane foaming agents used. Based on the material property data, the first polyurethane foaming agent, PU-1, has a maximum viscosity of approximately 1000 g/cm·s, making it the least viscous of the three materials. The allowable temperature range for the first polyurethane foaming agent (PU-1) is from 10 °C to 40 °C, which is narrower than that of the other agents. At a heating rate of 80 °C/min, PU-1’s viscosity sharply increases at 40 °C, indicating a rapid chemical reaction at this threshold, leading to swift hardening or solidification, which can result in incomplete fills. The second polyurethane foaming agent (PU-2) has a maximum viscosity of around 10,000 g/cm·s, positioning it in the middle of the three. Its acceptable temperature range, from 20 °C to 180 °C, is the widest. PU-2 exhibits a U-shaped viscosity trend with temperature; initially, viscosity decreases, but as the temperature approaches 160 °C, it rises sharply, triggering hardening or solidification. The third agent (PU-3) has the highest viscosity, approximately 100,000 g/cm·s, and its allowable temperature range is from 20 °C to 100 °C, placing it between the other two. Like PU-1, PU-3 shows a sharp increase in viscosity at elevated temperatures after an initial decrease, indicating accelerated reactions within a specific temperature range, resulting in a rapid viscosity increase.

**Figure 5:**
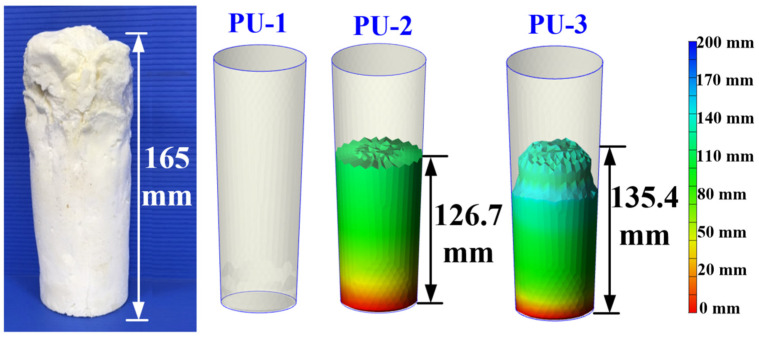
Foaming results of three polyurethane foaming agents.

To investigate which of the three polyurethane foaming agents used in this study produced foaming heights that most closely match experimental results, foaming experiments were conducted at a mold temperature of 60 °C. Figure 5 displays the foaming outcomes of these agents. The experimental results indicate that the actual foaming height of the polyurethane foaming agent is approximately 165 mm. In comparison, the simulation results show that the foaming height of PU-1 is approximately 0 mm, while PU-2 reaches a simulated height of 126.7 mm, and PU-3 reaches a simulated height of 135.4 mm. From these findings, four key observations were made: (a) Among the three simulated materials, PU-3 exhibited a foaming height closest to the experimentally observed height; (b) PU-1, with an operating temperature range between 10 °C and 40 °C, is highly temperature-sensitive. This causes it to solidify prematurely during the foaming process, preventing effective simulation; (c) PU-2 has a broad operational temperature ranging from 20 °C to 160 °C and shows a U-shaped viscosity profile relative to temperature. At a mold temperature of 60 °C, PU-2 achieves optimal fluidity, preventing excessive viscosity or premature solidification from blocking the gate. Additionally, its lower viscosity leads to a smoother foam surface; (d) PU-3, with an operational temperature ranging from 20 °C to 90 °C, reacts more rapidly at certain temperatures, resulting in a foaming height 8.7 mm higher than that of PU-2. However, the material distribution within the mold is uneven due to PU-3’s higher viscosity and limited fluidity, leading to noticeable fluctuations in the foam surface. Despite this, PU-3’s foaming behavior most closely resembles the experimental results. Therefore, based on these observations, PU-3 was chosen as the primary simulation material for further practical validation.

**Figure 6:**
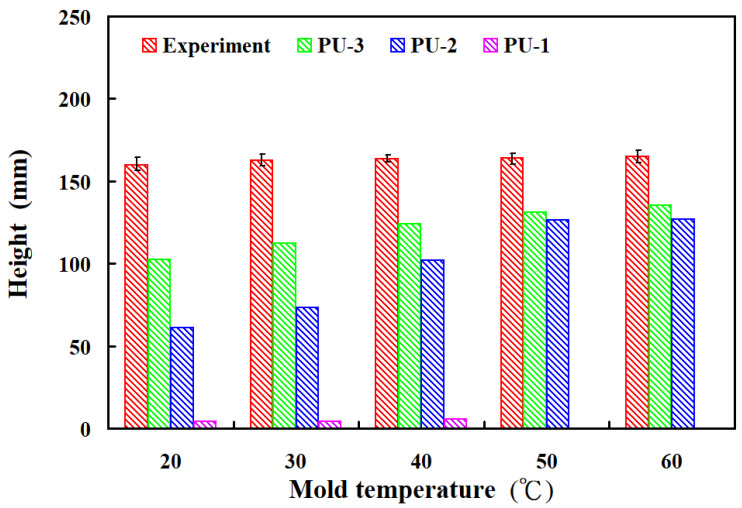
Foaming results of three polyurethane foaming agents at five mold temperatures.

**Figure 7:**
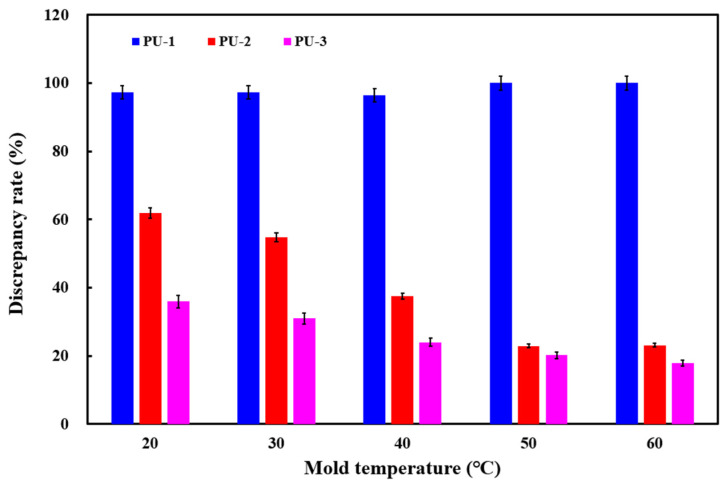
Discrepancy rate of foaming height of polyurethane foaming agents obtained from simulation and implementation of three polyurethane foaming agents.

Figure 6 shows the foaming results of three polyurethane foaming agents at five mold temperatures. The simulation results for PU-1 reveal foaming heights of approximately 4.25 mm, 4.25 mm, 5.73 mm, 0 mm, and 0 mm at the respective mold temperatures. For PU-2, the simulated foaming heights were around 60.87 mm, 73.37 mm, 102.15 mm, 126.50 mm, and 126.70 mm, respectively. The third polyurethane foaming agent, PU-3, exhibited foaming heights of approximately 102.36 mm, 112.10 mm, 124.25 mm, 130.98 mm, and 135.40 mm across the same temperature range. The average foaming heights observed in actual experiments for the five mold temperatures were approximately 159.87 mm, 162.37 mm, 163.53 mm, 164.07 mm, and 164.94 mm, respectively. The comparison between the simulation and the experimental results shows that the simulated foaming heights correspond to approximately 35.98%, 30.96%, 24.02%, 20.17%, and 17.90% of the experimentally obtained foaming heights for the respective mold temperatures. Figure 7 shows the discrepancy rate of the foaming height of polyurethane foaming agents obtained from simulation and implementation of three polyurethane foaming agents. The study reveals two significant findings: (a) among the three agents, PU-3 produced simulated foaming heights closest to the actual experimental results at all five mold temperatures, and (b) at a mold temperature of 60 °C, the simulated foaming height for PU-3 was the most accurate, with an error rate of only about 17.90%.

**Figure 8:**
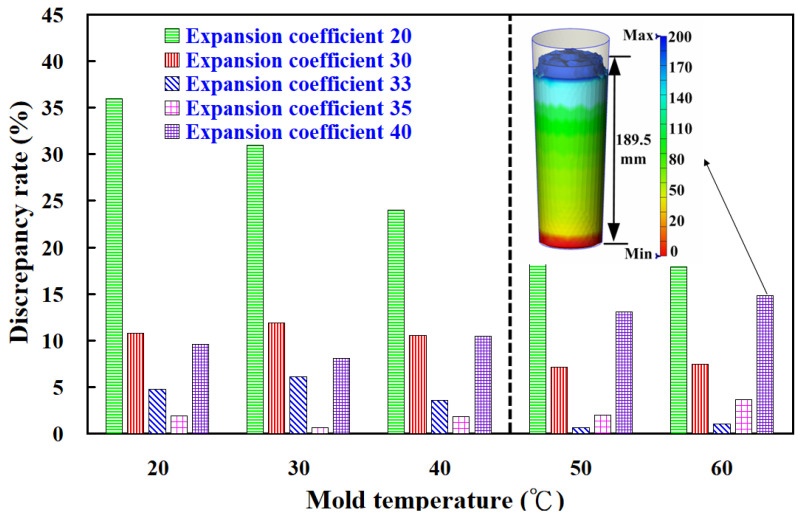
Discrepancy rate of foaming height of polyurethane foaming agent obtained from implementation and simulation using five different expansion coefficients within silicone rubber mold.

To explore the foam height of the polyurethane foaming agent PU-3 under different expansion coefficients, this study employed five distinct coefficients. Figure 8 presents the simulated foam heights at temperatures of 20 °C, 30 °C, 40 °C, 50 °C, and 60 °C using these coefficients in silicone rubber molds. When utilizing an expansion coefficient of 20, the error rates between the simulated foam heights and experimental values at 20 °C, 30 °C, 40 °C, 50 °C, and 60 °C were approximately 36%, 31%, 24%, 20.1%, and 18%, respectively. For an expansion coefficient of 30, the error rates were around 10.75%, 11.88%, 10.52%, 7.13%, and 7.45%. Using an expansion coefficient of 33, the respective error rates were 4.75%, 6.1%, 3.55%, 0.61%, and 1.03%. With an expansion coefficient of 35, the error rates were 1.94%, 0.62%, 1.83%, 2.01%, and 3.64%. Finally, for an expansion coefficient of 40, the error rates were 9.56%, 8.07%, 10.46%, 13.04%, and 14.85%. The study identified three key findings: (a) expansion coefficients of 33 and 35 yielded simulated foam heights that were closest to the experimental results; (b) for low-temperature foaming at 20 °C, 30 °C, and 40 °C, the expansion coefficient of 35 provided the most accurate foam height simulations for PU-3; and (c) for high-temperature foaming at 50 °C and 60 °C, the expansion coefficient of 33 produced simulations most aligned with the experimental outcomes.

**Figure 9:**
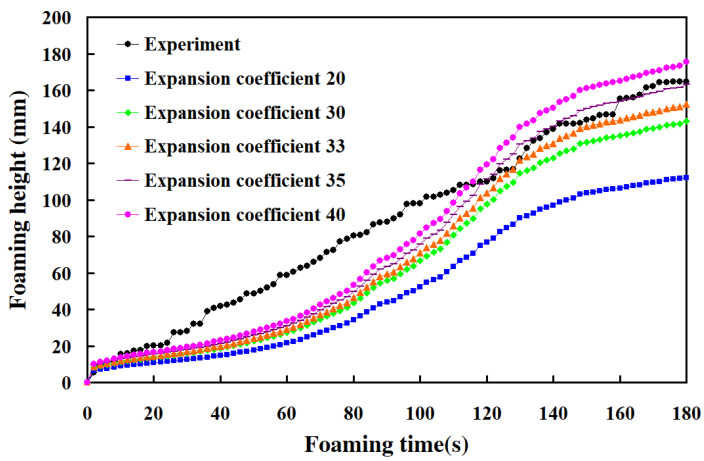
The relationship between the foam height and foaming time of polyurethane foaming agents.

**Figure 10:**
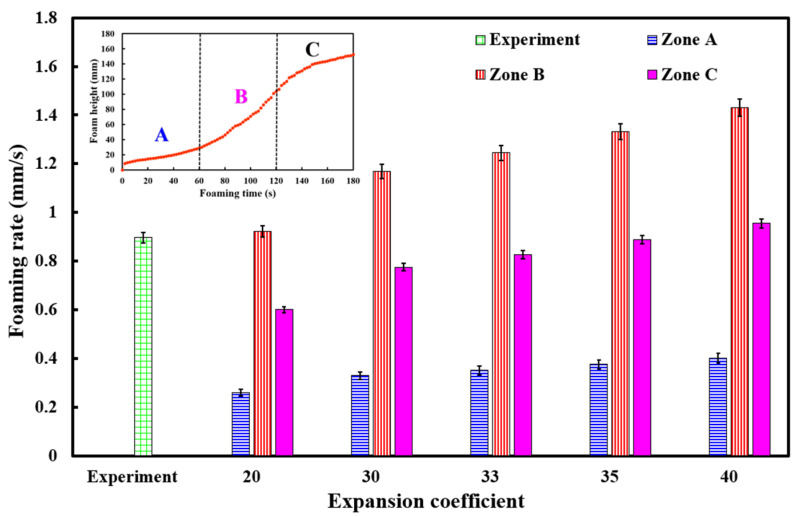
The foaming rate of polyurethane foaming agents.

To investigate the relationship between the foaming height and foaming time of polyurethane foaming agents, this study conducted experiments using the third polyurethane foaming agent in a silicone rubber mold at 30 °C under five different expansion ratios. The foaming time for the polyurethane agent was set to 180 s. Figure 9 illustrates the relationship between foaming height and foaming time, while Figure 10 presents the foaming rate. The results showed that with expansion ratios of 20, 30, and 33, the foaming height was lower than the experimental foaming height; with an expansion ratio of 35, the foaming height closely matched the experimental value, with an error of only about 0.83%. However, with an expansion ratio of 40, the foaming height exceeded the experimental value. Three key phenomena were observed. First, a linear relationship was found between foaming height and foaming time, with a foaming rate of approximately 0.9 mm/s. However, simulations using Moldex 3D indicated a non-linear relationship between foaming height and time, with three distinct foaming rates. Zone B exhibited the highest foaming rate, followed by Zone C, with Zone A showing the lowest rate. In Zone A, the foaming rates for expansion ratios of 20, 30, 33, 35, and 40 were 0.26, 0.33, 0.55, 0.35, and 0.37 mm/s, respectively. In Zone B, the rates were 0.92, 1.17, 2.56, 1.24, and 1.33 mm/s, respectively, while in Zone C, they were 0.6, 0.77, 1.67, 0.82, and 0.89 mm/s, respectively. Second, the foaming rate of the polyurethane agent increased with higher expansion ratios. Third, the foaming height of the polyurethane foaming agent in a silicone rubber mold at 30 °C could be predicted accurately using an expansion ratio of 35.

**Figure 11:**
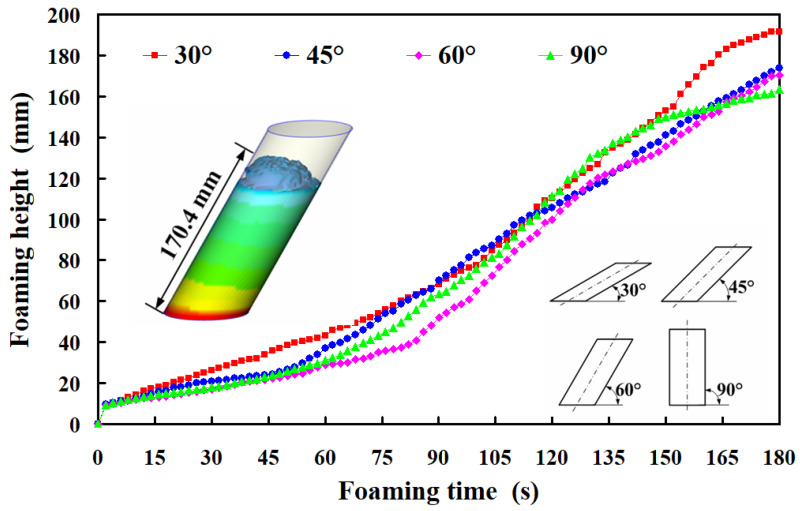
The relationship between the foam time and height of polyurethane foaming agents at four different foaming angles using simulation software.

**Figure 12:**
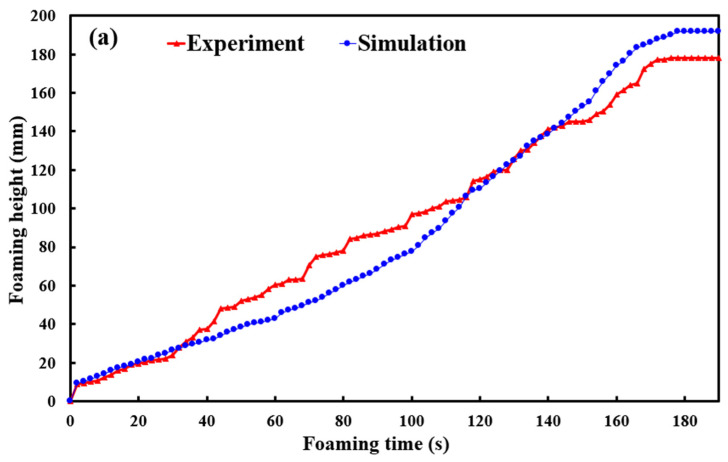

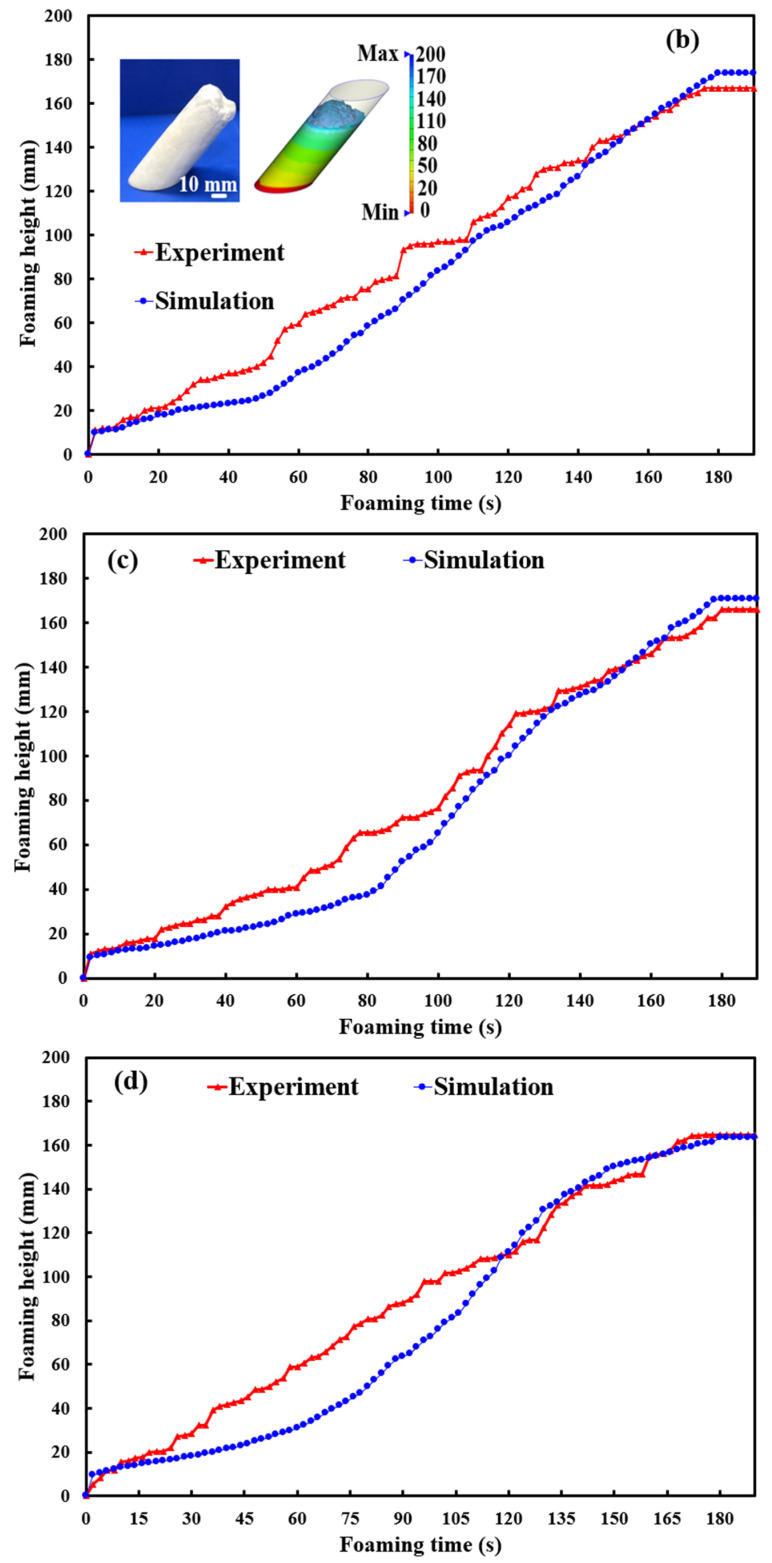
The relationship between experimental and simulated foaming time and height at four foaming angles of (**a**) 30°, (**b**) 45°, (**c**) 60°, (**d**) 90°.

To investigate the impact of various foaming angles on the foam height of polyurethane foaming agents, this study performed simulation foaming tests at a controlled temperature of 30 °C using silicone rubber molds. Four distinct foaming angles, i.e., 30°, 45°, 60°, and 90°, were evaluated to determine their effects on foam height. Based on simulation software, Figure 11 illustrates the relationship between the foam time and height of polyurethane foaming agents at four different foaming angles using simulation software. The simulation results revealed that, within a foaming duration of 180 s, the final foam heights for the respective angles were approximately 191.7 mm, 173.6 mm, 170.4 mm, and 163.4 mm. Analysis of these findings led to the observation of two significant phenomena: (a) a significant correlation exists between the foam height of polyurethane foaming agents and their foaming angle, and (b) the foaming rate is also influenced by the foaming angle. Specifically, a reduction in the foaming angle resulted in a more linear foaming curve, which correspondingly increased the final foam height, whereas an increase in the foaming angle was associated with a deceleration in the foaming rate. Figure 12 shows the relationship between experimental and simulated foaming time and height at four foaming angles of 30°, 45°, 60°, and 90°. The experimental findings indicate that the final foaming heights at angles of 30°, 45°, 60°, and 90° were 178 mm, 167 mm, 166 mm, and 165 mm, respectively. Two significant observations were made: (a) the foaming rate of the polyurethane foaming agent demonstrated consistent linear growth across varying angles; and (b) the simulation software accurately predicted the foaming rate, with strong agreement between the simulated and experimental results at both the initial and final stages of the foaming process.

**Figure 13:**
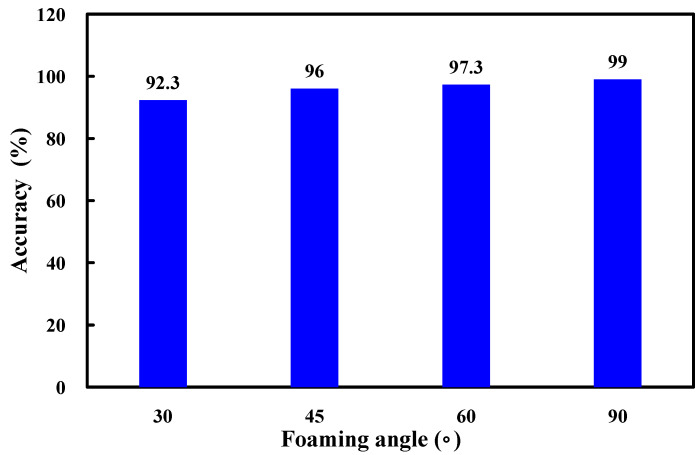
Accuracy of simulated foaming height at different foaming angles.

**Figure 14:**
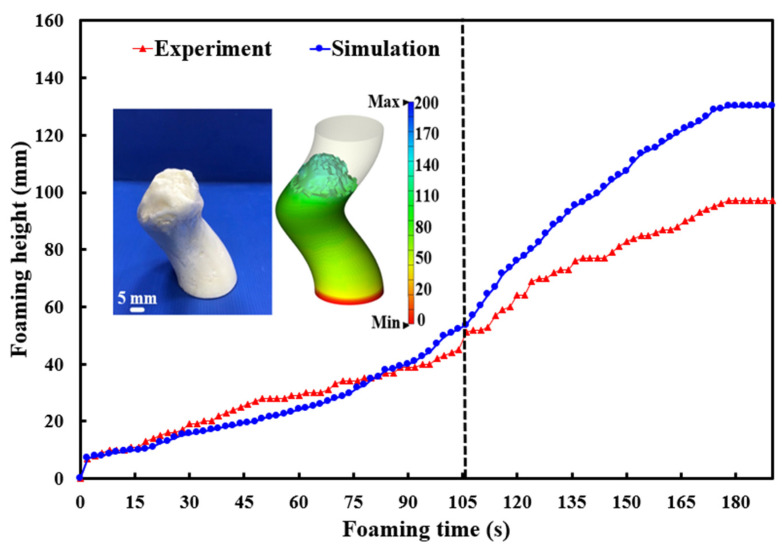
Results of 3D simulation and experimental study on polyurethane foaming agents.

Figure 13 shows the accuracy of the simulated foaming heights at different foaming angles. The results indicate that the accuracy rates of simulated foaming heights at foaming angles of 30°, 45°, 60°, and 90° were 92.3%, 96%, 97.3%, and 99%, respectively, with an overall average accuracy of approximately 96%. Two key observations emerged. One is that the accuracy of the simulated foaming height was lower at a foaming angle of 30°. The other is that the accuracy was highest at 90°. This disparity can likely be attributed to the significant influence of gravity on foaming behavior. At a foaming angle of 30°, the polyurethane foaming process becomes more complex, necessitating more sophisticated calculations by the simulation software. In contrast, at a 90° angle, the foaming material primarily experiences vertical gravitational forces [23], resulting in simpler foaming dynamics and consequently higher simulation accuracy. Therefore, a thermal expansion coefficient of 35 can be used in a silicone rubber mold at 30 °C to predict the foaming height of polyurethane foaming agents because the predicted foaming height of the polyurethane agent achieves an average accuracy of 96%. Based on the above experimental results, this study proposes two foaming mechanisms for polyurethane foam components. To further investigate the accuracy of predicting the foaming height of polyurethane foam agents using a silicone rapid mold at 30 °C with an expansion coefficient of 35, this study employed a 3D spiral component for testing. Figure 14 shows the results of the 3D simulation and experimental study on polyurethane foaming agents. In the experiments, the final foaming height reached 97 mm. However, simulations predicted that the foaming height of the polyurethane foam agent could reach 130 mm, with an accuracy rate of approximately 66%. The practical results and simulated foaming curves exhibited two distinct foaming trends. Specifically, the foaming curve displayed a relatively gradual increase from 0 to 105 s, followed by an almost linear growth pattern from 105 to 180 s. Due to the complex geometric structure of the 3D spiral component, the height growth of the foaming material within this space faced greater challenges, resulting in a significant discrepancy between the final simulated foaming height and the practical foaming height. This study observed two phenomena: first, there is a notable difference between the simulated results and the actual final foaming height of the 3D spiral space; second, the simulated and actual foaming curves exhibited considerable similarity.

**Figure 15:**
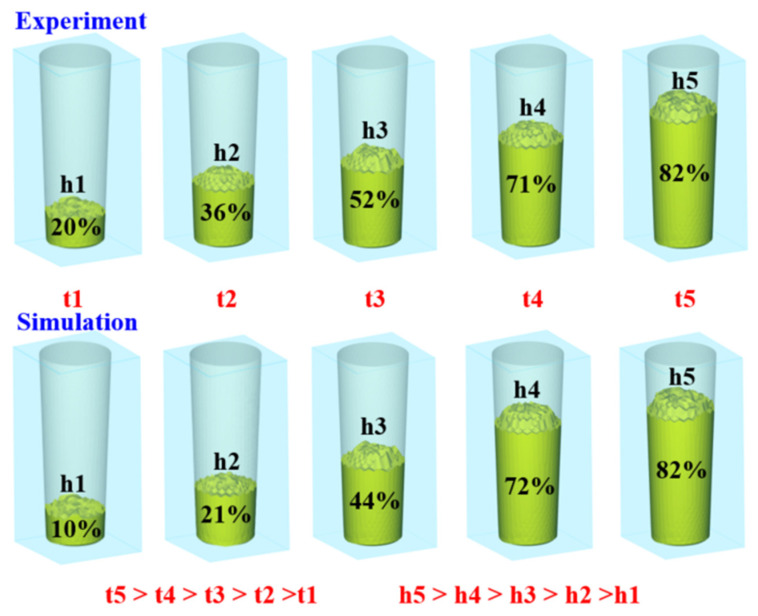
Foaming mechanism of 2D polyurethane foaming component.

**Figure 16:**
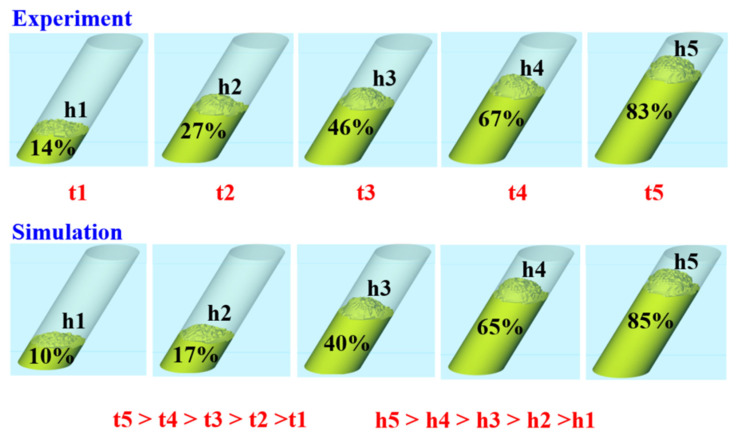
Foaming mechanism of 2.5D polyurethane foaming component.

**Figure 17:**
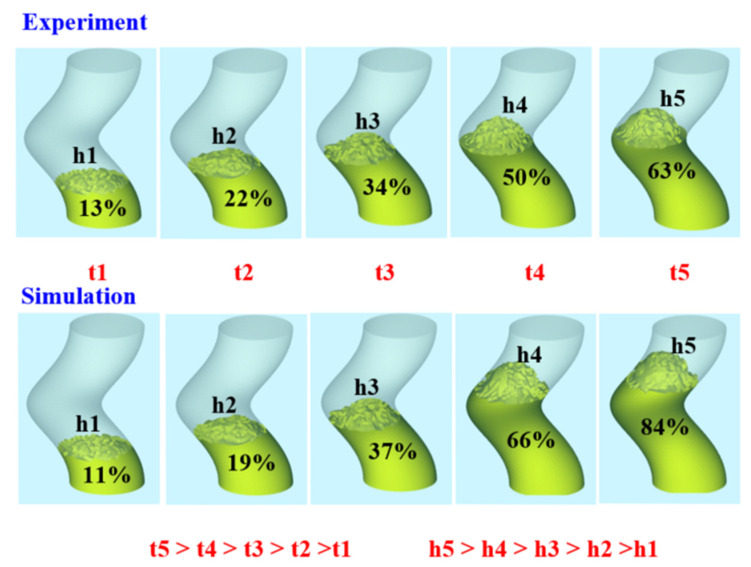
Foaming mechanism of 3D polyurethane foaming component.

Based on the aforementioned experimental results, this study proposes three mechanisms for the foaming of polyurethane foam components. Figure 15 shows the foaming mechanism of the 2D polyurethane foaming component. As the foam material begins to expand, it progressively rises, leading to a gradual increase in height. During the vertical foaming process, the lower layers of foam are subjected to both the weight and expansion forces of the upper layers due to the influence of gravity. As the expansion continues, the pressure exerted on the lower layers increases accordingly. Observations from actual foaming experiments demonstrate that foam height rises exponentially over time. In contrast, simulation results indicate that the simulated foam heights h1 and h2 are slightly lower than the actual values, while h3 matches the actual height. The simulated height h4 slightly exceeds the experimental result, and h5 is nearly identical to the actual height. Because the foaming mechanism of 2D polyurethane foam is primarily affected by vertical gravitational forces, the process remains relatively straightforward. However, this also means that, among different foaming angles, gravity has the most pronounced impact in the vertical orientation, resulting in the lowest final foam height at 90 degrees. Figure 16 shows the foaming mechanism of the 2.5D polyurethane foaming component. As the foam material begins to expand, it gradually moves upward, leading to an increase in height. During the foaming process at a 60-degree incline, the foam material at the lowest layer, under the influence of gravity, bears the weight and expansion pressure of the upper layers. As the material expands, the pressure exerted on the lower foam layer progressively increases. Observations from the foaming process indicate that the foam height increases geometrically over time. However, simulation results show that the simulated foam heights h1 and h2 are slightly lower than the actual foam height; the simulated height h3 closely matches the actual height; the simulated height h4 slightly exceeds the experimental results, and the simulated height h5 is very close to the actual foam height h5. The foam height at 60 degrees is more favorable for height increase because the 2.5D polyurethane foam specimen no longer expands vertically during the foaming process, and the gravitational effect at a 60-degree incline is less significant than at a 90-degree vertical expansion. Consequently, the final foam height at a 60-degree incline is slightly higher than that at a 90-degree vertical expansion. Figure 17 shows the foaming mechanism of the 3D polyurethane foaming component. As the foaming material begins to expand, it gradually moves upward, leading to an increase in height. During the foaming process in a spiral space, due to the influence of gravity, the foaming material at the lowest layer bears the weight and expansion pressure of the upper layers. As the material continues to expand, the pressure exerted on the lower layers progressively increases. Observations from the actual foaming process indicate that the foaming height rises exponentially over time. However, simulation results show that the simulated heights h1, h2, and h3 are consistent with the actual foaming heights. In contrast, the simulated height h4 is slightly higher than the experimental result, while the simulated height h5 significantly exceeds the actual foaming height. The complex geometry of the helical structure causes the foaming to develop along a curved path, introducing additional resistance and increasing the difficulty of expansion. Consequently, the final foaming heights in the practical experiments differ considerably from the simulated results.

**Figure 18:**
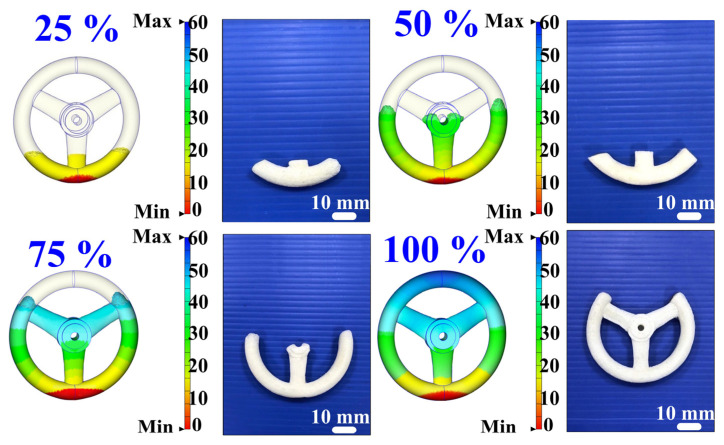
Simulation and experimental results of validation case one.

**Figure 19:**
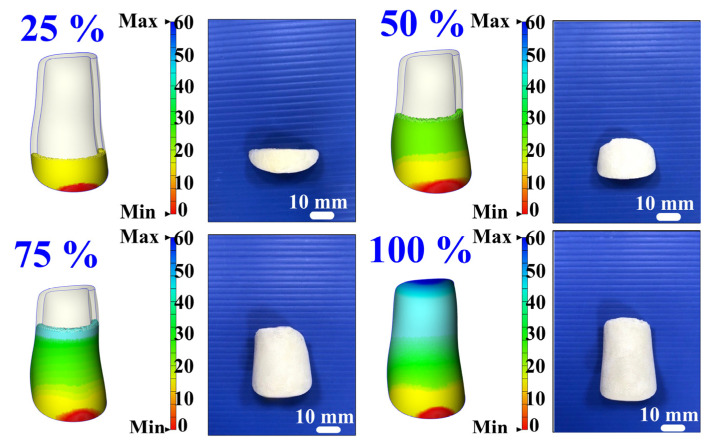
Simulation and experimental results of validation case two.

**Figure 20:**
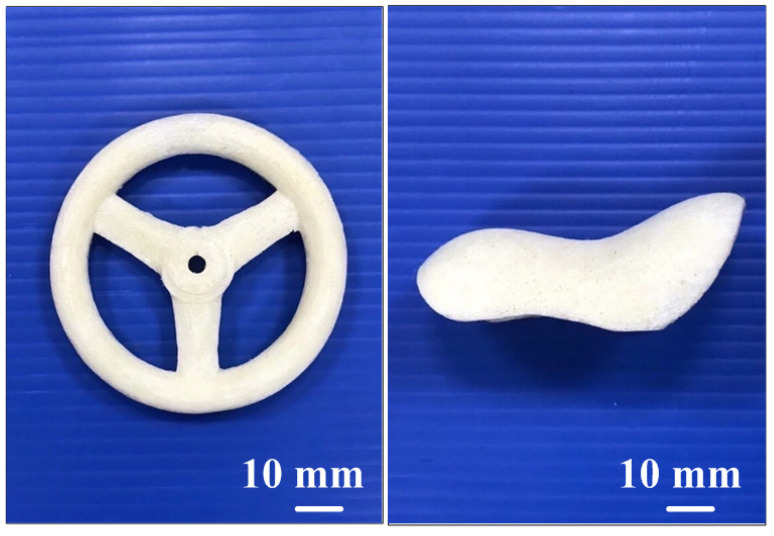
Final polyurethane foaming results of verification cases one and two.

To comprehensively investigate the filling behavior of polyurethane foaming agents in case one, this study performed simulations and experimental comparisons at fill levels of 25%, 50%, 75%, and 100%. Figure 18 shows the simulation and experimental results of validation case one. Both simulations and experiments utilized varying polyurethane foam weights—0.8, 1.75, 2.6, and 3.5 g—to achieve the designated fill levels. The simulation results indicated final foam heights of 14.7, 29.6, 44.3, and 60 mm for the 25%, 50%, 75%, and 100% fill levels, respectively, while the corresponding experimental results yielded foam heights of 10, 21, 33, and 44 mm. For fill rates of 25%, 50%, 75%, and 100%, the calculated accuracy rates were 53%, 59%, 66%, and 64%, respectively. Figure 19 shows the simulation and experimental results of validation case two. Based on the above results, this study identified two phenomena: (a) the foaming height trend of polyurethane foaming agents, as predicted by simulation software, aligns with experimental findings, and (b) the accuracy of foaming height predictions made by the simulation software for validation cases one and two is approximately 60.5% and 64.5%, respectively. Discrepancies between simulation and experimental results may stem from several factors: inaccurate material property parameters [24], improper boundary and initial conditions, limitations in numerical model precision, and coarse mesh division. Material parameters, like viscosity and density, if inaccurately set, may skew outcomes, while mismatches in boundary or initial conditions reduce reliability. Additionally, standard numerical models may inadequately represent non-linear behaviors like exothermic reactions and bubble dynamics. Optimizing mesh refinement and employing advanced models can mitigate these issues. These adjustments, including precise material property calibration, are essential to aligning simulation results closely with experimental data, enhancing predictive reliability. Figure 20 shows the final polyurethane foaming results of verification cases one and two. This study demonstrates that increasing the polyurethane foaming agent to 4 g, as opposed to the 3.5 g used in the simulation, achieves a fully formed polyurethane foaming product in both verification cases one and two. This adjustment highlights the sensitivity of the foaming process to agent quantity, where a 0.5 g increase optimally supports product formation. These findings suggest that precise calibration of the foaming agent amount is critical to achieving complete and consistent results in polyurethane foam production.

This study has focused on the innovative use of simulation technology to enhance the accuracy and efficiency of the polyurethane foaming process. Through the application of computer-aided engineering (CAE) simulations, foaming dynamics can be predicted with high precision, enabling improved control over the properties of the final product. This method not only minimizes material waste and reduces energy consumption but also enhances the overall quality and uniformity of polyurethane foam products. Consequently, the findings of this study align with the principles of green smart manufacturing. Green manufacturing emphasizes producing goods with minimal environmental impact by integrating practices such as resource efficiency, renewable energy utilization, and recycling [25]. It takes into account the entire product lifecycle, from design to disposal, to promote sustainability and reduce the overall carbon footprint. By embracing these principles, industries can balance economic growth with environmental responsibility, contributing to a more sustainable future. The outcomes of this research meet the criteria for green manufacturing and demonstrate significant potential applications in various industries. Moreover, these findings align with the Sustainable Development Goals (SDGs) 7, 9, 10, 11, and 12 [26]. Green smart manufacturing, which integrates green practices with advanced technologies such as artificial intelligence (AI) [27] and the Internet of Things (IoT) [28], further optimizes resource use and enhances productivity while reducing environmental impact [29,30,31,32]. By employing data-driven strategies and automation, this approach seeks to create efficient, sustainable manufacturing processes. Ultimately, green smart manufacturing aims to achieve both environmental sustainability and economic efficiency within the manufacturing sector.

## 4. Conclusions

This work has utilized computer-aided engineering simulations to enhance the accuracy and efficiency of the polyurethane foaming process, demonstrating the potential for widespread industrial applications and contributing to the goals of green manufacturing. The results of this research satisfy the criteria for green manufacturing and highlight substantial potential for applications across various industries. Furthermore, these findings align with SDGs 7, 9, 10, 11, and 12. The main conclusions from the experimental work in this study are as follows:

1. The results of this study demonstrate significant potential applications in industry, including construction for insulation, automotive noise reduction, packaging for cushioning, and lightweight structural components because the foaming height of PU-3 closely corresponds to the values obtained in the experiment. The experiment showed a linear relationship with foaming time at an approximate rate of 0.9 mm/s.
2. Moldex 3D simulations indicated a nonlinear relationship between foaming height and time, with three distinct foaming rates. Among the zones, Zone B showed the highest foaming rate, followed by Zone C, while Zone A exhibited the lowest rate.
3. A significant correlation between foam height and foaming angle has been revealed. Additionally, the foaming rate was influenced by the foaming angle, with smaller angles enhancing both foam height and foaming rate.
4. A thermal expansion coefficient of 35 can be used in a silicone rubber mold at 30 °C to predict the foaming height of polyurethane foaming agents. The predicted foaming height of the polyurethane agent achieved an average accuracy of 96% at four foaming angles.

## Figures and Tables

**Table 1:** Parameters used for simulating the polyurethane foaming process.

Items	Parameters
Mass of mixture injected into silicone mold (g)	18
Filling time of polyurethane foaming agent (s)	4.8
Mass flow rate (g/s)	3.75
Density of polyurethane foam (kg/m^3^)	1.087
Volume flow rate (m^3^/s)	3.45
Volume of the silicone rubber mold (cm^3^)	626.32
Foaming time (s)	180
Mold temperature (°C)	20, 30, 40, 50, 60
Ambient temperature (°C)	25

## Data Availability

The original contributions presented in this study are included in the article. Further inquiries can be directed to the corresponding author(s).
